# Implementation of Total Body Photography for Skin Cancer Early Detection: A Systematic Review

**DOI:** 10.1111/ijd.70679

**Published:** 2026-09-30

**Authors:** Nurul Amanina Binte Hussain, Frances Alice Ansah‐Sasraku, Pablo Fernández‐Peñas, Nicole Gillespie, Peter Hans Soyer, Ingrid Hickman, Monika Janda

**Affiliations:** ^1^ Frazer Institute, Dermatology Research Centre, The University of Queensland Brisbane Queensland Australia; ^2^ Faculty of Medicine and Health The University of Sydney Sydney New South Wales Australia; ^3^ Melbourne Business School The University of Melbourne Melbourne Victoria Australia; ^4^ Faculty of Health, Medicine and Behavioural Science The University of Queensland Brisbane Queensland Australia

**Keywords:** health system and services, skin cancer prevention, total body photography

## Abstract

Advances in imaging technologies, such as total body photography (TBP), and the increasing capabilities and use of artificial intelligence (AI) have the potential to enhance skin screening and skin cancer surveillance. However, limited research has examined how these technologies are implemented and integrated into clinical practice across different healthcare settings. This review examined the literature regarding the implementation and utility of TBP technologies over time. A systematic search identified 36 studies published from inception through to July 2025, describing TBP use and reported facilitators and barriers. Specifically, the review mapped TBP workflows, processes, workforce roles, contextual reporting, and evaluation of real‐world integration. Findings were synthesized using a narrative synthesis approach due to the heterogeneity in study designs, objectives, and reporting quality. The evidence base was largely implementation light, and no study applied a formal implementation science framework. Thirty‐six studies were included because they provided contextual information on TBP use. Only six studies reported any implementation‐related aspects, such as the feasibility of replicating TBP protocols across healthcare settings; user perceptions; and the practical use of TBP technologies and/or AI features. Reported technological advancements included digitally enabled and automated systems supporting skin lesion documentation, monitoring, and diagnostic decision‐making. Despite these technological shifts, descriptions of TBP work processes remained relatively consistent over time. Several studies reported on the feasibility of healthcare professionals from multiple disciplines to conduct TBP‐aided skin examinations. The findings highlight opportunities for TBP technologies to be scaled into a broader range of clinical and potentially resource‐constrained settings.

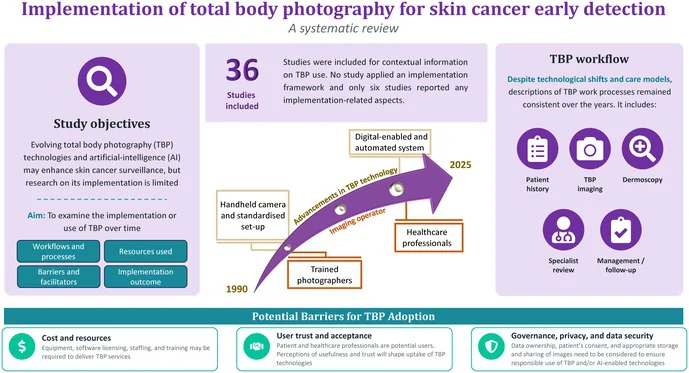

## Graphical abstract

This review maps the use of total body photography (TBP) for skin cancer early detection and prevention. It highlights the evolving TBP technologies used, personnel performing imaging, and potential care models employed. Despite this evolution, workflow processes remain broadly consistent, though variably sequenced across settings.

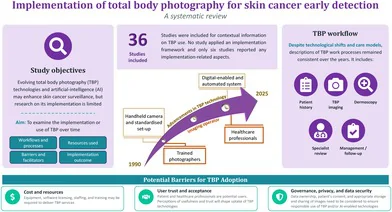

## 1 Introduction

Skin cancer is a public health burden worldwide. Globally, there were 331,722 cases and 58,667 deaths from melanomas, and 1.2 million cases and 69,416 deaths from non‐melanoma skin cancer (NMSC) in 2022 [1, 2]. NMSCs, now more commonly known as keratinocyte cancers, comprising basal cell carcinoma (BCC) and squamous cell carcinoma (SCC), are not routinely reported to cancer registries in Australia and globally, meaning that current estimates are likely to underestimate the true burden of disease [3]. Based on health service data, Australia and New Zealand had the highest age‐standardized incidence rates for NMSCs (138 per 100,000 persons) and melanomas (36 per 100,000 persons) in the world, almost double the regions with the second highest age‐standardized rate (North America and North Europe respectively) [1, 2]. An estimated two in three Australians are expected to be diagnosed with some form of skin cancer in their lifetime [4, 5]. Australia and New Zealand also had the highest age‐standardized mortality rate from melanoma (2.5 per 100,000 persons) [2].

The burden of skin cancer extends beyond the individual, having significant societal impact with a projected cost of approximately 378 and 337 billion international dollars between 2020 to 2050 respectively for NMSC and melanoma skin cancer globally [6, 7]. In Australia, the fiscal cost of diagnosing and treating melanoma and NMSCs was estimated to be one billion dollars [8, 9, 10, 11].

Despite high prevalence, skin cancers are preventable and treatable if detected early which can greatly reduce the morbidity and mortality. Public health campaigns promote skin cancer awareness [12], often recommending skin self‐examinations and presentation to clinician if concerned, especially for those at higher risk of developing skin cancer. Clinical skin examinations typically involve a visual inspection of the whole skin surface, frequently with the use of diagnostic imaging tools such as dermoscopy [8]. In specialized settings, these examinations may also incorporate clinical photography, and increasingly, total body photography (TBP).

In Australia, skin cancer detection and management are mostly done at primary care level, either in primary care or skin cancer clinics, followed by dermatologists, plastic surgeons and other specialists [13, 14]. About one third of Australians adults aged 45–69 years undergoes a clinical skin examination annually [15]. However, there are significant challenges that impede the accurate detection, monitoring, and appropriate management of skin cancer, such as the clinicians' skills and clinical workload [16, 17, 18].

Recent advancements in imaging technologies, such as TBP, offer opportunities to improve diagnostic accuracy as they enable clinicians to capture baseline images of the patient's entire skin surface and monitor or map lesions for abnormal changes over time. More recently, three‐dimensional (3D) TBP systems and the increasing use of artificial intelligence (AI) have expanded the potential of imaging‐based surveillance further. This prompted a growing number of studies evaluating the effectiveness of 2D and 3D TBP in detecting skin cancer. However, there remains a research gap in understanding how TBP technologies can be feasibly implemented and integrated across primary, secondary, and tertiary care settings.

Implementation refers to the processes through which interventions or technologies are adopted and integrated within healthcare setting [19]. Implementation outcomes of interest include acceptability (perception that technology is palatable among stakeholders), feasibility (extent that it can be successfully used in a setting), and appropriateness (perceived relevance of compatibility for a setting), and capture the fit, potential uptake, and sustained use of the technology within a given setting [20]. In contrast, clinical outcomes, such as thickness of melanoma detected or number of lesions to excise to detect a melanoma, evaluate the effectiveness of interventions [20, 21, 22]. This distinction matters because an intervention may be clinically effective yet fail to achieve its intended benefit if it is not implemented well, or does not fit for the setting, user, or recipient. Without guidance on effective implementation, the potential of TBP systems to strengthen skin cancer prevention and early detection is therefore unlikely to be fully realized. The aim of this review is to examine the implementation of TBP over time, guided by the following questions: (1) what workflows and processes were used to deliver TBP; (2) what resources were used to deliver TBP; (3) what barriers and facilitators influence the use of TBP; and (4) which implementation outcome measures were used or reported in studies of TBP use.

## 2 Materials and Methods

### 2.1 Search Strategy

This systematic review followed Cochrane guidelines and is reported according to Preferred Reporting Items for Systematic Reviews and Meta‐analyses (PRISMA). The comprehensive literature search was performed across four databases, PubMed, Scopus, Web of Science, and Embase, using combined key terms related to “Total Body Photography” and “skin cancer,” and Medical Sub‐Headings (MeSH) terms where applicable (Table S1). Search terms and strategies were reviewed, with inputs from a librarian to ensure alignment with the study objectives. The search was filtered to humans and English language and conducted without date restrictions to capture all relevant studies from inception to July 2025. Additional studies were identified through snowballing by screening the reference lists of identified literature. The study protocol was registered with the Prospective Register of Systematic Reviews (registration ID: CRD420251012909).

### 2.2 Selection of Articles

Studies were included if they described the implementation, integration, or utility of TBP technologies during skin examination for skin cancer. Due to the lack of study employing an implementation science approach, eligibility did not require implementation outcomes to be explicitly reported. Studies describing the clinical use of TBP were included where they provided insights into how TBP was used, by whom, in what setting, and under what conditions, consistent with this review's aim of mapping real‐world integration of TBP into clinical care. Studies were excluded if TBP technologies were used for other skin health and disease conditions, focused on the technical development and mechanism of TBP devices or its artificial intelligence (AI) components, inflammatory skin disease focus, or non‐human subjects.

All potentially relevant articles were imported to Covidence, a web‐based tool that facilitates screening of articles for systematic reviews, and EndNote, a reference management software. After removal of duplicates, three researchers (NABH, FAAS, XLS) independently screened and selected the articles via Covidence for inclusion. At the full‐text review phase, the initial inter‐reviewer agreement was fair (Cohen's *κ* = 0.35), reflecting differing initial interpretations of eligibility criteria. Discrepancies were resolved through discussion among the reviewers and clarifications of terminology ambiguity. Following these discussions, inter‐reviewer agreement improved to excellent (Cohen's *κ* = 0.86). Disagreements were resolved through discussion among team members and with supervisors.

### 2.3 Data Extraction and Analysis

A data extraction tool was developed in Microsoft Excel and piloted by two researchers to ensure comprehensibility, appropriateness, and ease of use. Data extracted included study details and design, study setting, devices used for TBP, resources required, and workflows and processes associated with TBP implementation. Following piloting, both researchers independently extracted data using the refined tool.

MeSH term for “whole body imaging” and a broad set of keywords related to total body imaging were used to ensure comprehensive coverage. TBP was not consistenly reported as “total body photography” across studies, with some literature describing specific devices, product names or programs, such as cameras, product, or programs. However, because these systems or devices involve capturing full body or regional images, they were identified through the broader imaging terms included in the search strategy. Where clarity was lacking, the researchers conducted targeted web searches of the devices used and discussed uncertainties with the research team to determine study inclusion and categorization for data extraction. Similarly, study settings were determined through web searches of the study site when not explicitly reported. Definitions from the Australian Institute of Health and Welfare were used to classify settings [23]. Primary care refers to the first point of contact within the health system, encompassing a broad range of health activities (e.g., health promotion, preventive health, and treatment of acute conditions). Secondary care is typically provided by specialists or facilities upon referral from a primary care provider, while tertiary care or hospital care involves highly specialized services [23].

Findings across the included studies were synthesized using a narrative approach, as this enabled the systematic summarization of heterogenous studies [24]. The researchers systematically reviewed and compared descriptions of study characteristics, TBP technologies, resources involved, and workflow integration or work processes, to identify patterns, divergences, and contextual influences on TBP use.

### 2.4 Quality Assessment

Quality of included articles was assessed using the Joanna‐Briggs Institute tools for observational and experimental studies [25, 26]. However, final assessment scores were not used as a basis for study exclusion as this review focuses on qualitative information to understand how TBP was integrated and used across various care settings, and not on effect sizes or other quantitative information.

## 3 Results

A total of 644 articles were identified across the four databases, with one additional article identified from reference screening. After removal of duplicates (*n* = 287), 358 articles were screened for eligibility. Of these, 322 articles were excluded as they did not meet the inclusion criteria, such as the lack of focus on skin cancer or not describing the clinical use of TBP for early skin cancer detection (Figure S1). Thirty‐six articles met the inclusion criteria and were included in the review.

### 3.1 Study Characteristics

The studies employed a wide range of methodological approaches, including observational studies [27, 28, 29, 30, 31, 32, 33, 34, 35, 36, 37, 38, 39], descriptive or cross‐sectional studies [40, 41, 42, 43, 44, 45], comparative studies [46], cohort studies [47, 48, 49, 50, 51, 52, 53, 54, 55, 56, 57, 58], cost‐effective study [59], case–control studies [60], non‐randomized controlled trial [61], and randomized controlled trials [62]. Study size ranged from 70 to 1955 participants (Table 1). All included studies recruited individuals at risk of skin cancer or patients undergoing pigmented lesion monitoring within a clinical setting. Only six studies specifically reported some aspects of TBP implementation in their study [42, 49], or implementation‐related outcomes (e.g., user acceptability and perceptions) [36, 48, 50, 51], but none of the studies incorporated a formal implementation framework.

**TABLE 1:** Characteristics of included studies.

Author, year, (country)	Study objective	Study setting^a^	Study period	Study size	Results
Arzberger, 2016 (Austria)	Compare medical recommendation of in‐person care versus teledermatology	Tertiary level, Outpatient clinic at teaching hospital	2009–2013	70	No. of lesions excised/recommended for excision = 62 No. of melanoma = 10 Benign to malignant ratio = 5.2 *Teledermatology vs. in‐person* Diagnostic accuracy = 67% vs. 80% Management decision = 70% vs. 65%
Banky, 2005 (Australia)	Determine the incidence of new, changed, and regressed nevi using baseline TBP and dermatoscopy	Tertiary level, Private dermatology practice	1992–1998	309	Incidence of new pigmented lesion = 277 per 1000 person years Incidence of melanoma = 19 per 1000 person years Benign to malignant ratio = 2.8% with Breslow thickness < 0.75 mm = 80%
Cerminara S. E., 2023 (Switzerland)	Investigate clinical performance of 3D TBP, 2D TBP, dermatologist, and augmented AI	Tertiary level, Teaching hospital	2021	143	No. of lesions excised = 75 Excision per patient, mean (SD) = 1.4 (0.6) No. of melanoma‐in situ (%) = 72 (50%) No. of metastatic melanoma (%) = 2 (1%) *Diagnostic performance (sensitivity, specificity)* Dermatologist = 90%, 92% 2D TBP = 70%, 40% 3D TBP = 90%, 65% Augmented intelligence = 90%, 86%
Chan, 2024 (Australia)	Clinical predictors of malignancy in lesions	Tertiary level, Referral centre at teaching hospital	2017–2021	NA	No. of invasive melanoma (%) = 26 (43%) Breslow thickness, median = in situ % with Breslow thickness < 1 mm = 97% (*n* = 58)
Chousakos E. 2022 (USA)	Investigate distribution of acquired melanocytic neoplasms	Tertiary level, Specialized cancer center	2017–2020	311	No. of lesions classified as melanomas (%) = 72 (1%) No. of lesions classified as nevi (%) = 5036 (99%)
Deinlein 2020 (Austria)	Evaluate usefulness of two‐step algorithm in early melanoma detection	Tertiary level Teaching hospital	2011–2015	214	No. of lesions excised (%) = 35 (69%) No. of melanomas (%) = 11 (22%) No. of invasive melanomas (%) = 10 (91%) Breslow thickness, mean = 0.44 mm NNE = 4.6
Drugge E. D 2021 (USA)	Report effect of using transportable TBP to provide intermediate examinations	Secondary level Dermatology practice	May–Nov 2020	1674	Ratio of melanoma in situ and invasive = 1.7: 1 Biopsies per skin cancer = 1.52 No. of participants who used mobile TBP (%) = 591 (35%) No. of participants who used telemedicine appointment (%) = 357 (61%)
Drugge, R. 2010 (USA)	Evaluate screening with serial total body photography	Secondary level, Dermatology practice	Aug–Dec 2009	64	No. of lesions of concern = 394 No. of changed lesions (%) = 306 (78%) No. of new lesions (%) = 36 (9%) Overall patient satisfaction with process = 73% Ease of following direction = 81%
Gellrich, 2024 (Germany)	Benefits of three‐step imaging programme	Tertiary level, Teaching hospital	2021–2024	410	*Procedures done in imaging programme vs. outpatient* No. of excisions = 61 vs. 195 No. of melanoma (%) = 16 (26%) vs. 7 (4%) No. of melanoma in situ (%) = 8 (13%) vs. 6 (3%) No. of invasive melanoma (%) = 8 (13%) vs. 1 (1%) Breslow thickness, range = 0.3–0.9 mm vs. 0.7 mm NNE = 2.4 vs. 23.3
Goessinger, 2024 (Switzerland)	Perspectives of patients and dermatologists on use of AuI	Tertiary level, Teaching hospital	Jan–Dec 2021	205	No. of excisions (%) = 78 (38%) No. of malignant lesions (%) = 17 (22%) *Dermatologist perspectives (n = 205)* Believe AuI can improve diagnostic performance = 94% Perceive AI to be added benefit in cases of uncertainty = 36% Beginner dermatologists who placed more trust in AI assessment in cases of uncertainty = 76% *Patient perspectives* Trust in 2D TBP vs. 3D TBP (*n* = 201) = 93% vs. 92% Preference for AuI with 2D TBP vs. 3D TBP (*n* = 199) = 22% vs. 61%
Goodson A. G., 2011 (USA)	Compare TBP diagnostic capabilities with digital dermoscopy	Tertiary level Specialized cancer centre	2004–2009	1076	*On follow‐up* No. of biopsy = 275 biopsy in 467 patients Biopsy rate per patient = 0.59 No. of biopsy done, motivated by photographic comparison (%) = 168 (61%) No. of melanomas (%) = 12 (4.4%) No. of MIS (%) = 7 (58%) No. of invasive melanoma (%) = 5 (42%) Invasive melanoma depth = 0.19–0.65 mm No. of melanomas that arose de novo (%) = 9 (75%)
Greenwald E., 2021 (New Zealand)	Assess effectiveness of telemedicine service	Primary level Skin cancer clinic	2015–2016	1389	No. of melanomas (%) = 260 (17%) No. of melanoma diagnosed at initial visit vs. follow‐up visit (%) = 88 (34%) vs. 172 (66%) No. of melanoma in situ, overall (%) = 101 (74%) No. of invasive melanoma, overall (%) = 36 (26%) Breslow thickness, median = 0.50 mm % melanomas with Breslow thickness < 0.8 mm = 92% Benign to malignant ratio = 4.9: 1 Number needed to biopsy = 6.0
Guitera, 2021 (Australia)	Sustainability and expansion of surveillance programme	Tertiary and primary level, Outpatient HRC at teaching, major referral clinics, and skin cancer clinic	2012–2020	593	Number of excisions = 1513 No. of melanomas (%) = 171 (11%) Benign to malignant ratio = 0.8 No. with Breslow thickness > 1 mm (%) = 7 (4%) No. of new melanomas most likely to be detected by clinician with TBP aid (%) = 54 (32%) No. of new melanomas most likely to be detected by DD monitoring (%) = 50 (29%)
Hobelsberger, 2023 (Germany)	Evaluate performance of 3D TBP in detecting and diagnosing NMSC	Tertiary level, Teaching hospital	2021–2022	129	*Performance of 3D TBP vs. dermoscopy* Sensitivity for BCC = 73% vs. 79% Specificity for BCC = 77% vs. 82% Diagnostic accuracy for BCC = 75% vs. 80% Sensitivity for SCC = 81% vs. 74% Specificity for SCC = 75% vs. 84% Diagnostic accuracy for BCC = 76% vs. 82%
Hobelsberger, 2024 (Germany)	Benefits of three‐step imaging programme	Tertiary level, Teaching hospital	2021–2024	600	No. of excisions = 265 No. of malignant melanomas = 23 No. of MIS = 14 No. of invasive melanomas = 9 No. of lesions with tumor thickness < 1 mm = 9
Hodgkinson, 2025 (UK)	Effectiveness of nurse‐led surveillance	Tertiary level, Hospital network	2016–2024	1680	No. of excisions = 345 No. of MIS (%) = 20 (6%) No. of invasive melanomas (%) = 21 (6%) No. with Breslow thickness < 0.8 mm (%) = 20 (95%) Number needed to biopsy = 8.41
Horsham, 2022 (Australia)	Feedback from TBP study participants	Tertiary level, Outpatient HRC at teaching hospital	Over 3 years	149	*Participant feedback on TBP use (n = 149)* % participants stating that they trust TBP at 18‐month vs. 36‐month = 69% vs. 70% % participants stated they were comfortable at 18‐month vs. 36‐month = 71% vs. 61% Consider using technology in future = 94% Perceived 3D‐TBP to be a useful tool = 99%
Jahn, 2022 (Switzerland)	Diagnostic accuracy of AI app vs. AI TBP	Tertiary level, Teaching hospital	Jan–June 2021	114	No. of lesions excised (%) = 61 (5%) No. of melanoma = 6 *Patient perspectives (n = 114)* Trustworthiness of 2D TBP = 90% Trustworthiness of 3D TBP = 90% Favor combination of dermatologist and 3D TBP for risk assessment = 57%
Kovalyshyn I., 2011 (USA)	Compare melanoma characteristics and detection patterns between new and established patients	Tertiary level, Specialized cancer centre	1998–2008	394	Benign to malignant ratio (10‐year period) = 5.4 No. of lesions detected with TBP (%) = 110 (31%) *Melanoma in established (n = 325) vs. new (n = 169) patients* ^b^ % in situ lesions = 70% vs. 57% % invasive melanoma = 91% vs. 43% Breslow thickness, mean = 0.45 mm vs. 0.82 mm
Lallas 2020 (Greece)	Identify risk factors and assess usefulness of TBP in detecting SPM	Tertiary level Referral center in a hospital	2013–2018	977	No. of new primary melanomas = 52 No. of in situ melanomas (%) = 35 (67%) No. of invasive melanomas (%) = 17 (33%) Breslow thickness, mean = 0.49 mm No. of lesions excised (%) = 25 (48%)
Lindsay D. 2025 (Australia)	To compare costs and health effects of 3D TBP and SDDI vs. usual care for early detection of melanoma.	Tertiary level Outpatient clinic at teaching hospital	2018–2021	309	*Intervention (n = 157) vs. control (n = 152)* No. of melanoma excised (%) = 38 (36%) vs. 69 (64%) No. of MIS excised (%) = 20 (83%) vs. 34 (79%) Mean OOP costs for skin tumor services = $333 vs. $395 Mean QALYs over 2 years = 1.84 vs. 1.84
Moloney, 2014 (Australia)	Impact of skin examinations with TBP and SDDI	Tertiary level, Outpatient HRC at teaching hospital and major referral clinics	2006–2011	311	Number of excisions = 770 No. of malignant lesions (%) = 291 (38%) Benign to malignant ratio = 1.6 No. of primary melanomas during follow‐up = 61 Breslow thickness, median = 0.33 mm (TBP detected) or in situ (SDDI detected)
Nathansohn, 2007 (Israel)	Describe set‐up of specialized lesion clinic	Secondary level Specialized skin cancer clinic	2005–2006	895	No. of lesions recommended for excision (%) = 236 (5%) No. of melanomas = 7
Nguyen J. 2022 (Australia)	Identify and compare dermoscopic features of developing melanomas and benign nevi	Secondary level Dermatology clinic	2007–2019	94^c^	No. of melanomas = 89 No. of invasive melanoma (%) = 34 (38%) Breslow thickness, median = 0.35 mm % of melanomas associated with a nevus = 45% Change in size was seen equally in both melanomas (87%) and benign nevi (83%). Asymmetric change was significantly more common in melanomas and symmetric change was significantly more common in benign nevi^d^
Rademaker, 2010 (New Zealand)	Assess ability to detect melanoma early	Tertiary level, Teaching hospital	2005–2007	100	No. of melanomas = 100 No. of MIS (%) = 48 (48%) No. of invasive melanoma (%) = 52 (52%) Breslow thickness, mean = 0.87 mm Breslow thickness < 0.75 mm = 69%
Reiter O. 2022 (USA)	Describe long‐term evolution of melanocytic nevi	Tertiary level, Specialized cancer center	Prior to 2005	106	New nevi at f/u, mean = 2.6 No. of patients with increasing nevus count (%) = 62 (58%) No. of nevi which grew in diameter by at least 25% during f/u = 1416 (49%) No. of nevi imaged using TBP = 2890
Risser, 2007 (USA)	Determine effect of TBP on biopsy rates	Secondary level, Specialized skin cancer clinic	1998–2003	128	*With TBP* No. of biopsies, mean = 1.89 (53 biopsies, 28 patients) No. of melanomas = 0 *Without TBP* No. of biopsies, mean = 1.76 (51 biopsies, 29 patients) No. of melanomas = 3 Breslow thickness of each melanoma = 0.3 mm, 0.44 mm, and 1.1 mm
Salerni G. 2012 (Spain)	Benefits of two‐step method (TBP and DD)	Tertiary level, Teaching hospital	1999–2008	618	No. of lesions for follow‐up (mean) = 11,396 (18.4) No of lesions excised (mean) = 1152 (1.9) No. of new or changed lesions excised (%) = 373 (32%) No. of melanomas = 98 (9%) Benign to malignant ratio = 10.7: 1 No. of MIS (%) = 53 (53%) No. of invasive melanomas (%) = 45 (46%) Breslow thickness, median = 0.5 mm
Saura 2021 (Spain)	Determine number of biopsies needed to diagnose melanoma and their clinical characteristics	Tertiary level Teaching hospital	2002–2016	152	No. of biopsies performed (mean) = 99 (0.7) No. of malignant melanoma (%) = 13 (13%) No. of MIS (%) = 8 (62%) No. of invasive melanomas (%) = 5 (38%) Breslow thickness, mean = 0.19 mm Mean time to diagnose = 4.7 years Benign to malignant excised ratio = 6.6: 1
Shriner^e^ 1990 (USA)	Describe TBP technique for patient with DNS	Tertiary level, Medical center facility	NA	NA	NA
Soyer H. P., 2025 (Australia)	Clinical outcome with TBP and SDDI	Tertiary level, Outpatient HRC at teaching hospital	2018–2024	314	*Overall* No. of excisions = 1527 excisions in 226 participants No. of melanomas (%) = 67 (4%) Breslow thickness = only 1 with thickness > 0.8 mm *Intervention (n = 122) vs. control (n = 104)* No. of excisions = 905 vs. 622 excisions No. of malignant lesions (%) = 280 (31%) vs. 189 (30%) No. of melanomas (%) = 24 (3%) vs. 43 (7%) No. of MIS (%) = 20 (2%) vs. 34 (5%) No. of invasive melanoma (%) = 4 (0.4%) vs. 9 (1%) Benign to malignant ratio = 2.2 vs. 2.3 NNE for melanoma = 3.2 vs. 3.3
Soyer H.P. 2023 (Australia)	Improve understanding of epidemiology and natural history of melanocytic naevi in adults	Tertiary level Teaching hospital	2016–2020	164	No. of participants referred to own doctor (%) = 108 (56%) for 250 lesions of concern No. of melanomas = 6 Benign to malignant ratio = 2: 1 NNE = 3: 1
Strunck, 2020 (USA)	Examine melanoma diagnoses and role of TBP monitoring	Tertiary level, Specialized cancer centre	2004–2013	1955	No. of MIS (%) = 51 (42%) No. of invasive melanomas (%) = 70 (58%) Breslow thickness, median = 0.33 mm No. of melanomas diagnosed following TBP = 121 No. of new melanomas detected by TBP (%) = 11 (92%) No. of changing melanomas detected by TBP (%) = 65 (83%)
Tan A. 2022 (USA)	Preliminary review of US‐based melanoma surveillance service	Secondary level, Melanoma surveillance service	NA	240	No. of melanoma = 23 (7%) Breslow thickness, median = 0.22 mm No. of melanomas with Breslow thickness that are invasive (%) vs. in situ = 10 (56%) vs. 8 (44%) Benign to malignant ratio = 9.7: 1 Number needed to biopsy = 13.4
Watts, 2015 (Australia)	Cost‐effectiveness of surveillance programme	Tertiary level, Outpatient HRC at teaching hospital	2012–2013	102	No. of melanomas detected from excised lesions (%) = 3 (10%) *Estimated costs* Total health system costs^f^ = AUD 227,649 Mean health system costs per patient per year = AUD 882 OOP costs for a set of total body photographs = AUD 34 per item
Winkler J. K. 2024 (Multiple countries)	Investigate performance of an automated total body mapping algorithm to detect clinically relevant melanocytic lesions	Tertiary and primary level, Specialized skin clinic and teaching hospitals	2021–2022	236	Total number of lesions excised (%) = 49 (16%) Total number of malignant lesions (%) = 14 (29%) *At follow‐up* No. of CRMLs = 334 No. of CRMLs detected by automated lesion detection (%) = 323 (97%) No. of new lesions detected = 13 No. of lesions changed = 24 *User perception (n = 233)* Increased safety, Agree = 93% Technology is trustworthy, agree = 92% Improve performance of dermatologists, agree = 88% Completely replace dermatologist examinations, disagree = 66% Accepted longer examination times, agree = 71%

Abbreviations: 2D TBP, Two‐dimensional Total Body Photography; 3D‐TBP, Three‐dimensional Total Body Photography; AI, Artificial Intelligence; AuI, Augmented Intelligence; BCC, Basal Cell Carcinoma; CRML, Clinically relevant melanocytic lesions; DD, Digital dermoscopy; DNS, Dysplastic Naevus Syndrome; HRC, High Risk Clinic; MIS, Melanoma in situ; MPM, Multiple Primary Melanomas; NA, Not available, information is not clearly reported; NMSC, Non‐melanoma Skin Cancer; NNE, Number needed to excise; OOP, Out‐of‐pocket; QALY, Quality‐Adjusted Life Years; SCC, Squamous Cell Carcinoma; SDDI, Sequential Digital Dermoscopy Imaging; SPM, Secondary Primary Melanomas; TBP, Total Body Photography.

^a^ Tertiary level includes hospitals and hospital networks, Secondary level includes specialists or facilities requiring referrals, and primary level includes facilities with a broad range of health services from promotion to treatment of acute conditions.

^b^ Study compares outcomes between regular and new patients.

^c^ Study reports descriptive statistics by melanoma analysis group and benign nevi analysis group.

^d^ Statistically significant finding at *p*‐value < 0.05.

^e^ Paper described how they take total body images at their practice.

^f^ Health system costs include overheads and capital (e.g., Equipment, software and licenses, medical equipment, hospital floor space) of a high risk clinic during a 12‐month period.

Less than one‐third of studies reported on workflow steps (*n* = 11) [30, 31, 35, 38, 39, 41, 42, 43, 46, 58, 62], process duration (*n* = 12) [30, 37, 38, 42, 45, 46, 48, 50, 55, 56, 58, 62], or audit or quality assurance processes (*n* = 2) [42, 49]. In terms of resourcing (Table 2), few studies reported staffing roles, especially image operators (*n* = 14) [30, 35, 39, 41, 42, 44, 45, 46, 51, 52, 53, 54, 57, 63], training requirements (*n* = 5) [30, 35, 39, 42, 46], number of images produced (*n* = 14) [27, 31, 33, 37, 38, 41, 42, 43, 45, 46, 47, 49, 51, 62], and IT systems used for imaging and/or image storage (*n* = 18) [31, 32, 34, 35, 38, 39, 40, 41, 42, 43, 44, 46, 49, 50, 54, 55, 59, 62]. Only four studies reported on costs, with two reporting user costs [45, 47], and two reporting on the service costs [56, 59]. In terms of governance and privacy, only two studies reported privacy safeguards, such as applying password encryption for image storage systems [38, 42].

**TABLE 2:** Overview of TBP‐related information extracted from the included studies.

Author, year	TBP device	Clinical setting^a^	Follow‐up period	No. of images	IT system	Imaging duration	Imaging operator
Arzberger E., 2016	TBP—Digital camera MoleMap program, Dermoscopy	Tertiary	NA	24 per patient	Images uploaded to program's centralized server with remote access	30–90 min per patient	Trained specialist nurse, called melanographer or skin imaging technician, or resident
Banky J., 2005	TBP—Camera, Dermoscopy	Tertiary	3, 6, or 12 months	14 per patient	NA	NA	NA
Cerminara S., 2023	TBP—FotoFinder	Tertiary	NIL	20 per patient	NA	10–15 min	NA
	3D TBP—Vectra WB360 DD			92 per session^c^		Imaged within a few seconds	
Chan W., 2025	TBP^b^ SDDI	Tertiary	Regular intervals over 1–5 years	NA	NA	NA	NA
Chousakos E., 2022	3D TBP—Vectra WB360	Tertiary	NA	92 per session^c^	VAM Vectra software to outline anatomical boundaries and calculate surface area	NA	NA
Deinlein T., 2020	TBP—FotoFinder, SDDI	Tertiary	NA	20 per patient	NA	NA	NA
Drugge E., 2021	TBP—Melanoscan	Secondary	NA	65 per session	Data was stored in a proprietary electronic health record and image database	NA	Trained technician
Drugge R., 2010	TBP—Melanoscan	Secondary	12 months	56 per patient	Data stored in a developed software system.	3 min for image acquisition	Trained technician
Gellrich F., 2024	3D TBP—Vectra WB360, DD, RCM	Tertiary	3, 6, or 12 months	92 per session^c^	NA	NA	NA
Goessinger E., 2024	TBP—FotoFinder	Tertiary	NA	NA	NA	2 h for overall study duration	NA
	3D TBP—Vectra WB360, DD			92 per session^c^			
Goodson A., 2011	TBP—Digital camera	Tertiary	6 or 12 months	27 per patient	Images stored in a software on the institution's server and on CD‐ROM	15 min photography session	NA
Greenwald E., 2021	TBP—MoleMap NZ	Primary	NA	NA	Images stored and forwarded to offsite dermatologist	NA	Trained registered nurse referred to as a melanographer
Guitera P., 2021	TBP—Camera, Polartechnic, MoleMap, and FotoFinder, SDDI	Primary and Tertiary	3 or 6 months	12–24 per patient	Images stored in a database. Copy of images were stored in a DVD/USB for the patient	NA	NA
Hobelsberger S., 2023	3D TBP—Vectra WB360	Tertiary	NIL	92 per session^c^	NA	NA	NA
Hobelsberger S., 2024	3D TBP—Vectra WB360, DD, RCM	Tertiary	3, 6, or 12 months	92 per session^c^	NA	NA	NA
Hodgkinson T., 2025	TBP—FotoFinder, SDDI	Tertiary	6 or 12 months	NA	NA	Imaging done within 30 min consult	Trained nurse specialist
Horsham C., 2022	3D TBP—Vectra WB360, Dermoscopy	Tertiary	6 months for 3 years	92 per session^c^	Vectra software	Imaged within a few seconds, and avatar created in 10 min	NA
Jahn A. S., 2022	TBP—FotoFinder ATBM	Tertiary	NIL	20 per patient	NA	NA	Medical students and study nurse
	3D TBP—Vectra WB360, Dermoscopy			92 per session			
Kovalyshyn I., 2011	TBP^b^ SDDI	Tertiary	3 and/or 6 months	NA	NA	NA	Clinicians
Lallas A., 2011	TBP^b^ DD	Tertiary	4 or 6 months	NA	NA	NA	Clinician specialist
Lindsay D., 2025	3D TBP—Vectra WB360	Tertiary	Every 6 months in 2 years	92 per session^c^	Vectra imaging system and integrated software	NA	NA
Moloney F. J., 2014	TBP—MoleMap and Polartechnic, SDDI	Tertiary	3 or 6 months	12–24 per patient	Patient data is stored in a purpose‐built database. Images were also stored on DVD for patients	NA	NA
Nathansohn N., 2007	TBP—Digital camera, DD	Secondary	3, 6, or 12 months	20 per patient	Images stored in a dedicated program. Images were stored on a CD for patients	NA	NA
Nguyen J., 2023	TBP—Digital camera MoleMap NZ, SDDI	Secondary	NA	NA	Lesion changes were measured using an incorporated feature on MoleMap software	NA	NA
Rademaker M., 2010	TBP—Digital camera MoleMap NZ, SDDI	Tertiary	NIL	NA	Images stored and managed in a proprietary software connected to a remote server	NA	Trained nurse referred to as a melanographer
Reiter O., 2022	TBP—Derma‐Graphix, 3D TBP—Vectra WB360	Tertiary	NA	92 per session^c^	Vectra software	NA	Trained clinical photographer
Risser J., 2007	TBP^b^	Secondary	NA	NA	NA	NA	Experienced technicians
Salerni G., 2012	TBP—MoleMax system, DD	Tertiary	3, 6, or 12 months	NA 126.3 body maps per patient (mean)	NA	Examine patients within 30–45 min	Dermatoscopy expert
Saura P. S., 2021	TBP—MoleMax system, DD	Tertiary	NA	16.9 per patient (mean)	NA	NA	NA
Shriner D. L., 1990	TBP—Digital camera	Tertiary	NIL	85 per adult patient	NA	20–30 min session	Medical photographer
Soyer H., 2025	3D TBP—Vectra WB360, SDDI	Tertiary	6 months in 2 years	92 per session	VECTRA software	Imaged within a few seconds, and avatar created in 10 min	NA
Soyer H., 2023	3D TBP—Vectra WB360, Dermoscopy	Tertiary	6 months for 3 years	92 per session^c^	VECTRA software	Imaged within a few seconds, and avatar created in 10 min	NA
Strunck J. L., 2020	TBP^b^	Tertiary	3 to 6 months	NA	Images were archived using Mirror software	NA	NA
Tan A., 2022	TBP—MoleSafe USA program, Dermoscopy	Secondary	12 months	NA	Images stored on telemedicine platform	NA	Trained nurse referred to as a melanographer
Watts C., 2015	TBP—MoleMap and Polartechnic, SDDI	Tertiary	6 or 12 months	NA	NA	Mean consult 31–40 min, includes TBP	NA
Winkler J. K., 2024	TBP—FotoFinder	Primary and tertiary	3 to 12 months	NA	NA	NA	NA

Abbreviations: 3D‐TBP, Three‐dimensional Total Body Photography; DD, Digital dermoscopy; NA, Not available, information is not clearly reported; RCM, Reflectance Confocal Microscopy; SDDI, Sequential Digital Dermoscopy Imaging; TBP, Total Body Photography.

^a^ Tertiary level includes hospitals and hospital network, Secondary level includes specialists or facilities requiring referrals, and primary level includes facilities with broad range of health services from promotion to treatment of acute conditions.

^b^ Study did not provide device description or specification.

^c^ VECTRA WB360 machine has 92 cameras and takes simultaneous images to create a single three‐dimensional avatar.

### 3.2 TBP in Skin Cancer Surveillance

The earliest paper describing the use of TBP was published in 1990. Initially, TBP was performed using handheld cameras, mostly by specialist medical photographers, requiring specific photographic equipment such as reflex lenses and controlled environmental set‐up, such as standardized lighting to ensure that images were consistent and of high quality (Table 2) [43, 44, 45, 47]. Some of these practices are reflected in a best‐practice guideline for structured and reproducible imaging protocols published in 2018 [64].

After 1999, studies started to report the use of a standardized digital imaging system, such as MoleMax (MoleMax Systems, Vienna, Austria), to facilitate TBP (Figure 1) [33, 58]. Since then, studies described a number of proprietary systems or programmes, including MoleMap (MoleMap Ltd., Auckland, New Zealand) [31, 32, 39, 44, 46, 49, 56], MoleSafe USA (which is based on MoleMap) [35], Melanoscan [41, 42], Polartechnics (which was delisted in 2012) [31, 49, 56], and FotoFinder (FotoFinder System GmbH, Bad Birnbach, Germany) [27, 30, 36, 37, 48, 51]. Similar to handheld photography, these systems also take images in a two‐dimensional (2D) plane; however, they differ in the use of handheld versus scaffolding fixated cameras that allow for simultaneous image capture. Increasing access to automated imaging systems improved the reproducibility of photo‐taking, which enabled more accurate comparison of baseline and updated images [38, 41, 55].

**FIGURE 1:**
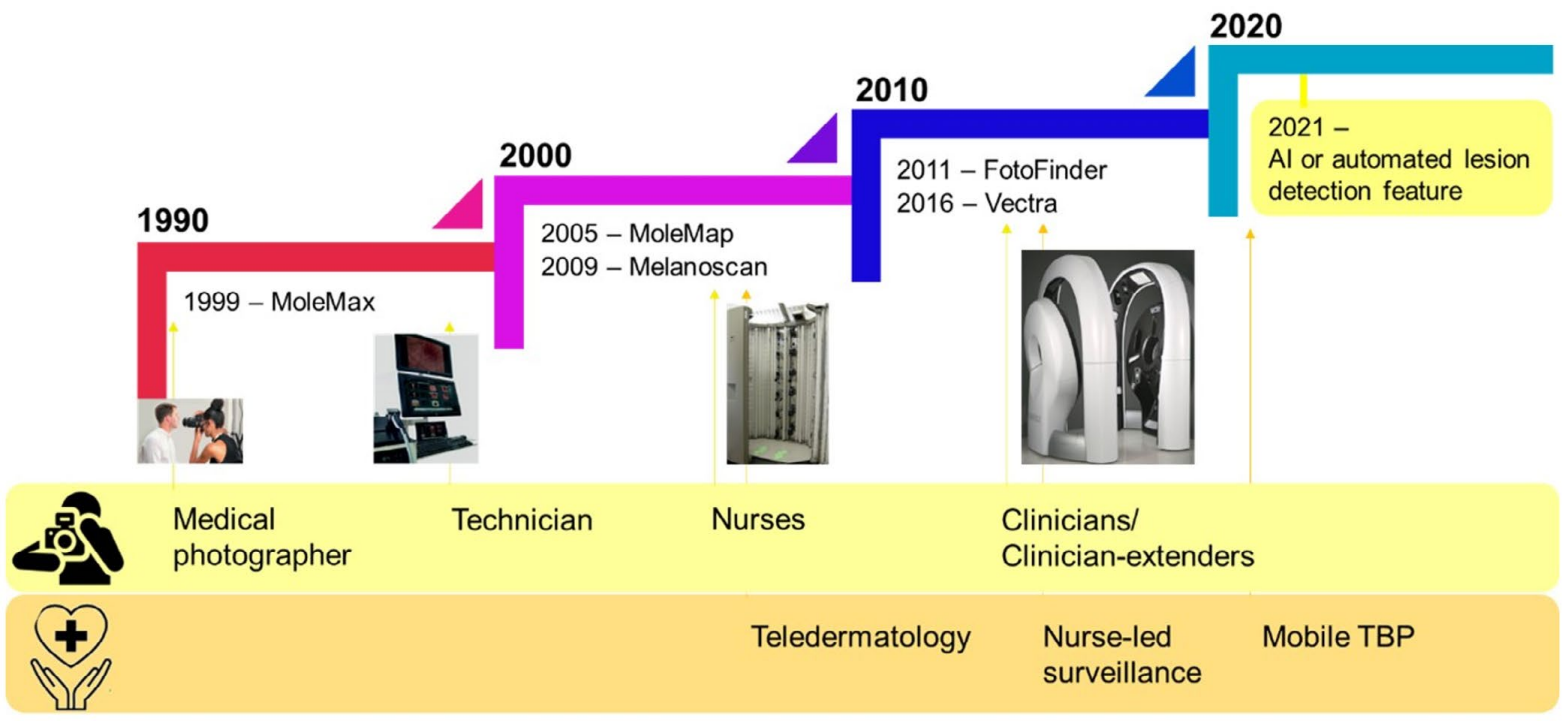
Evolution of total body photography (TBP) use for skin surveillance.

The transition from 2D to three‐dimensional (3D) TBP also marked a significant technological advancement. The earliest study included in this review that reported the use of 3D TBP, which was mainly the Vectra WB360 (Canfield Scientific, Parsippany, NJ, USA), was initiated in 2016. This 3D TBP machine also uses a camera array for automated and simultaneous image capture. However, its unique feature is software to create a 3D avatar of the patient, which allows clinicians to closely inspect the skin and lesions, including location on the body, size, color, and border features [29, 37].

A total of 24 studies reported the use of TBP [27, 30, 31, 32, 33, 34, 35, 36, 38, 39, 41, 42, 43, 44, 45, 46, 47, 49, 52, 53, 56, 57, 58, 60], with a further eight studies specifically 3D‐TBP [28, 29, 40, 50, 55, 59, 61, 62], and four studies used both 2D‐ and 3D‐TBP (Table 2) [37, 48, 51, 54]. Studies using TBP were conducted to assess skin cancer characteristics or epidemiology (*n* = 5) [32, 33, 47, 52, 60], evaluate impact, usefulness, effectiveness, or cost‐effectiveness of a surveillance program (*n* = 16) [27, 30, 31, 34, 35, 36, 38, 39, 41, 42, 44, 46, 53, 56, 57, 58], and sustainability or scalability of a surveillance programme (*n* = 1) [49]. The remaining two studies described the use of 2D TBP in their clinic [43, 45]. Studies using 3D TBP assessed its diagnostic capability, clinical outcome, or epidemiology or distribution of melanocytic naevi (*n* = 3) [40, 55, 62], impact, effectiveness, benefits of imaging techniques or cost‐effectiveness in surveillance (*n* = 4) [28, 29, 59, 61], and patient experience (*n* = 1) [50]. Studies using both 2D‐ and 3D‐TBP investigated diagnostic accuracy and performance or user perspectives on the use of integrated AI (*n* = 3) [37, 48, 51], and described the long‐term evolution of melanocytic naevi among high‐risk participants (*n* = 1) [54]. Despite variation in study focus, most studies employed TBP as a longitudinal monitoring tool, reporting a study duration of at least 3 years. In contrast, only six studies were conducted within a year.

### 3.3 Use of Artificial Intelligence

Traditional use of TBP for skin cancer surveillance has relied on the manual interpretation and comparison of serial images to monitor and identify changing skin conditions [28, 29, 32, 42, 43, 47, 49, 53, 60]. The number of images captured using TBP can range from 12 to 85 images per patient, which health professionals can use to compare baseline and follow‐up images to detect changes [27, 31, 33, 38, 41, 42, 43, 45, 46, 47, 51]. Such manual review of images can be time‐intensive and require considerable effort from the health professionals.

Digital imaging systems used in studies from 2004 onwards enabled the use of software functionalities that support image comparisons and enhance lesion tracking and evaluation. These include capabilities to zoom or scale images [29, 38], outline anatomical boundaries and calculate a surface area of a lesion [28], measure the size of a lesion [32, 54], and obtain detailed location of lesions [40]. Additional features, such as color registration, time‐lapse montage, and flicker techniques, may also support in visualizing changes to the lesion over time [42].

Nine studies in this review reported the use of AI or automated lesion detection features, with most studies initiated from 2021 onwards (Figure 1) [28, 29, 36, 37, 48, 50, 51, 54, 61]. AI components had been integrated into TBP imaging platforms, such as the convolutional neural network‐based MoleAnalyzer Pro (FotoFinder Systems GmbH, Bad Birnbach, Germany) and DEXI (Dermoscopy EXplainable Intelligence) (Canfield Scientific, Parsippany, NJ, USA), which are part of a 2D‐ and 3D‐TBP device respectively [37, 48, 51]. Jahn et al. and Cerminara et al. reported the application of a study‐defined cut‐off score for AI‐based malignancy risk assessment [37, 51]. Other studies reported the use of an automated feature for skin lesion counting, rating of size, color, or border irregularities, mole mapping, and lesion detection [28, 29, 36, 50, 53, 54, 55].

### 3.4 Personnel in TBP Image Acquisition

The personnel responsible for image acquisition in TBP has evolved considerably alongside technological advancements. Two studies reported the use of medical photographers to perform manual TBP (Table 2) [45, 54]. Earlier TBP techniques, involving the use of handheld cameras with specific apparatus, required highly skilled and medically trained photographers to ensure that images were anatomically correct, consistent and had good magnification and high‐resolution [45, 47]. Additionally, three studies described the set‐up of their photography studio, guiding patient positioning and poses, and systematic capture of body sectors, to ensure the acquisition of reproducible and comparable images [43, 45, 47]. Three studies reported the use of technicians to perform TBP, although it remains unclear whether their roles were aligned with the medical photographers [41, 42, 57]. In one study, technicians were also responsible for reviewing image quality, with oversight and feedback provided by supervising dermatologists [42].

Ten studies documented health professionals performing TBP or skin examinations which involve TBP. More specifically, nurses were trained to perform TBP, with most taking on the role of a melanographer [30, 35, 39, 44, 46, 51]. Tan et al. described the provision of an eight‐week training ‘melanographer’ programme, delivered by MoleMap programme, to prepare nurses [35]. Additionally, some care models require clinical nurse specialists to undertake periodic dermoscopy training to perform their roles in the given setting [30, 39]. Fewer studies reported that medical students or clinicians conducted TBP or captured clinical images [51, 53, 59]. Notably, many studies in this review did not clearly specify the qualifications of the personnel responsible for TBP image acquisition [27, 28, 29, 31, 32, 33, 34, 36, 37, 38, 40, 43, 47, 48, 49, 50, 55, 56, 59, 60, 61, 62].

### 3.5 Care Settings and Models

Across the included studies, 27 studies were conducted in tertiary‐level settings [27, 28, 29, 30, 31, 33, 34, 37, 38, 40, 44, 45, 46, 47, 48, 50, 51, 52, 53, 54, 55, 56, 58, 59, 60, 61, 62], six in secondary‐level settings [32, 35, 41, 42, 43, 57], one in primary‐level setting [39], and two in mixed settings that spanned tertiary and primary care (Table 2) [36, 49]. There was no clear chronological trend in the adoption of TBP across these settings.

Tertiary care settings predominantly comprised teaching hospitals, specialized cancer centers, or referral cancer centers. Specialized clinics and hospital care are characterized by access to a wide range of resources, including highly skilled dermatologists or specialized clinicians, specialized nurses, and pathology departments [32, 36, 43, 46, 49, 60]. However, some studies also reported that larger hospitals may employ or train a higher proportion of junior or less experienced clinicians, which could influence patient assessments [37, 48]. Watts et al. reported that labor costs were a major contributor to overall healthcare expenditure in skin cancer surveillance, due to the use of specialized expertise at the tertiary level [56].

One study explored TBP implementation in primary care, recognizing its potential to enhance skin cancer surveillance while reducing hospital health service costs [49]. Guitera et al. observed similar excision rates and favorable benign melanocytic to melanoma ratios when their structured surveillance protocol, aided by TBP and sequential digital dermoscopic imaging (SDDI), was replicated at other centers including a primary care clinic [49]. However, several studies also noted challenges that may limit the feasibility of implementing TBP at the primary care level, including the costs and time required to conduct skin examinations [28, 56]. Although some TBP systems were described to be time‐efficient, this may be attributed to the availability of trained and specialized healthcare professionals to conduct TBP and dermoscopic assessments [29, 37, 38, 48, 50, 61, 62].

Several studies evaluated the benefits of skin cancer surveillance programmes [28, 31, 42], including nurse‐led models [30, 39], and asynchronous teledermatology approaches [35, 39, 41, 46, 55, 59, 62]. One study uniquely assessed the use of mobile TBP services during the COVID19 pandemic [41]. The ability to store images within secured or proprietary platforms, such as MoleMap, and manage them via a remote server accessible from any authorized computer, supports the teledermatology model in skin cancer surveillance (Table 2) [44, 46]. Nurse‐led surveillance program involved clinical nurse specialists to conduct dermoscopic examination, though image review and clinical recommendations were still performed by consultant dermatologists [30, 39]. Tan et al. and Greenwald et al. also reported having melanographers take patient history and perform full skin examinations, in addition to TBP, supervised by a medical clinician. Images taken were then stored and forwarded to pigmented lesions experts for review and clinical management recommendations [35, 39]. Similarly, Soyer et al. described a telereview process in which dermatologists provided clinical recommendations based on submitted images [55].

Across the included studies, it was evident that despite differences in TBP technology used, the workflow was relatively consistent across the different care models (Table 3, Figures S2, S3). Patients at risk of skin cancer risk were considered or referred to skin cancer surveillance with TBP imaging [28, 30, 33, 38, 39, 40, 42, 43, 47, 49, 52, 54, 56, 57, 58, 60]. Procedures typically involved collection of medical history, TBP, skin examination, and dermoscopic imaging [27, 28, 29, 31, 33, 35, 36, 37, 38, 39, 41, 42, 43, 44, 46, 47, 48, 49, 51, 52, 54, 55, 56, 58, 60, 61, 62]. Thereafter, a dermatologist or clinical specialist would review the images and advise further management of suspicious lesions or appropriate follow‐up intervals [27, 28, 30, 31, 32, 33, 35, 37, 38, 39, 42, 43, 44, 46, 47, 48, 49, 50, 51, 52, 55, 56, 58, 59, 60, 61, 62]. Variability in workflow occurred in the sequence of when TBP or skin examinations were conducted, whether dermoscopy was performed during the TBP encounter, and whether the images were reviewed in real‐time or asynchronously. This variation may be attributed to site‐specific conditions. For example, Drugge et al. reported an unexpected benefit of reviewing the images before skin checks as it allowed treatment procedure to be prepared in advance, avoiding the need for patients to return on as separate visit [42].

**TABLE 3:** TBP‐related work processes identified across included studies.

Theme	Processes
Patient eligibility	TBP is recommended or offered to patients with risk of melanoma or skin cancer
Patient referral	Patients are referred by their general practitioners and specialists or self‐refer themselves to undergo TBP services
Consultation	Collect patient's medical history and skin cancer risks
Preparation of machine	TBP equipment or machine is calibrated before the first image session. Ensure appropriate exposure and magnification, for 2D TBP, or cameras are aligned and functioning, for 3D TBP
Patient Communication	Risks and benefits of TBP were explained to patients
	Instructions given to patients to undress and stand at the assigned spot in preparation for TBP. Note: Some studies provide disposable undergarments for patients to put on, if required
Imaging	Patients position themselves following an audio and/or visual guide for imaging
Image storage	TBP images were stored in a dedicated body mapping program, proprietary software, and/or secured server. Some allow for healthcare providers to remotely access the images using an authorized computer
Images provided to patients	Copies of the images may be provided to the patients for self‐skin examination
Clinical examination	Total body cutaneous examination performed using the naked eye and/or handheld dermoscopy
	Any naevus that meets the criteria of being suspicious will be marked by the dermatologically trained clinician on the 3D avatar and/or images
Follow‐up and further management	Lesions may be sent to a dermatologist or specialized clinician for review and/or management plan
	Patients with lesions with concerning features will be referred to their regular clinician for management
	Lesions with concerning features will be recommended for further surveillance or for excision and/or biopsy
	Reports are communicated with stakeholders: referring and consulting physicians, patients, caregivers, and insurance companies
During follow‐up session	Total body cutaneous examination performed
	TBP images taken during follow‐up sessions will be compared with the images taken at baseline
Technical issues	Patients can be rescheduled in the event of a technical issue, affecting function of TBP machine
	Images were stored in a CD‐ROM which will be used in the event of server problem
Audit processes	Some sites were regularly audited to make sure all parameters (study protocol) were standardized
	Dermatologists reviewed the images taken by other healthcare provider and maintained a feedback loop to improve imaging role

Abbreviations: 2D TBP, two‐dimensional total body photography; 3D‐TBP, three‐dimensional total body photography; TBP, total body photography.

While dermatologists remain central to image interpretation and clinical decision‐making, many procedural tasks, including history‐taking, TBP, and skin examinations, can seemingly be delegated to other trained healthcare professionals, supporting broader implementation across diverse care settings. Consequently, TBP can be incorporated into a range of care models, including nurse‐led services, teledermatology pathways, or standalone imaging services that operate independently of dermatology visits.

### 3.6 Benefits and Challenges of Implementing TBP Technologies

Across studies, TBP technologies were described as having the potential to enhance clinician's ability to monitor an individual's skin for new or changing lesions and support early detection of skin cancer (Table 4) [27, 28, 30, 33, 34, 38, 42, 47, 49, 52, 60]. Newer digital imaging systems improve the reproducibility of images, enabling direct comparison between baseline and follow‐up images [28, 38, 41, 42, 55, 62]. Several studies reported a number needed to excise (NNE) or number needed to biopsy (NNB) ranging from 2.4 to 8.4 to detect a melanoma [27, 28, 30, 39, 55, 62], with only one study reporting a NNB of 13.4 [35]. Moreover, where reported, a higher proportion of melanomas detected had a Breslow thickness of < 1 mm [30, 35, 38, 44, 47, 49, 52, 53, 60, 62]. These findings suggest that TBP contributes to detecting melanomas at an earlier and thinner stage. Another benefit is that TBP protocols and set‐ups can be standardized and implemented across different healthcare settings, even independently of dermatological visits [28, 39, 41, 42, 49, 55, 56, 62].

**TABLE 4:** Benefits and challenges to adopting or implementing TBP technologies.

	TBP^a^	3D TBP^b^
**Benefits**		
Improve ease of monitoring and early detection	Supports monitoring and early detection of skin cancer as baseline and follow‐up images are compared for changes to the skin. Reduce potential inaccuracies due to recall [27, 28, 30, 33, 34, 38, 42, 47, 49, 52, 60]	Creates single patient three‐dimensional avatar, which provides Supporting information of lesion compared to 2D images. Reduce potential inaccuracies due to overlapping images enabling continuous naevus count [29, 37]
	Some TBP systems enable reproducible imaging due to simultaneous image capture and/or standardized set‐up and processes [38, 41, 42, 55]	Allows automated lesion detection and sequential comparison, supporting identification of changes to the skin [28, 55, 62]
	Improves patient follow‐up compliance, due to overall benefits and patient's satisfaction towards TBP services [38, 61]	
Support self‐skin examination	Copies of TBP images can be provided to patients for self‐skin examination^c^ [31, 34, 43, 49]	
Improve health service delivery	TBP systems can be more time‐efficient, compared to serial digital dermoscopy [38]. Particularly, TBP system which uses multiple cameras for simultaneous imaging [42]	3D TBP system is more time‐efficient than manual 2D TBP system, as imaging can be done simultaneously and ease of image documentation [29, 37, 38, 48, 50, 61, 62]
	Supports teledermatological services as image acquisition procedure can be done separately, from dermatologist visits [28, 39, 42, 49, 55, 62]	
	TBP systems and/or protocol recommendations can be adapted to various settings [56]. Some TBP system can also be converted to a mobile platform, improving access to care [41, 42, 56]	
	Most TBP system requires less physical space than 3D TBP system^d^ [36]	
**Challenges**		
Limited lesion visibility, capture, and detection	Some body areas cannot be adequately captured/imaged, due to occlusion (e.g., skin folds, hair or lack of exposure to the cameras (e.g., scalp, sole of feet)) [36, 42, 45, 47, 54]	Some body areas cannot be adequately captured/imaged, due to occlusion (e.g., skin folds, hair or lack of exposure to the cameras (e.g., scalp, sole of feet)), or image resolution is lower than dermoscopic images [28, 29, 38, 50, 61]
Patient physical ability		Patients with mobility issues may not assume the standardized patient postures [29, 49]
Resources are needed in providing services	Some TBP systems require specific equipment (e.g., cameras with accessories for adequate magnification) and/or set‐up (e.g., lights, markings) to ensure consistency in imaging [27, 45, 58]	Novel 3D TBP is currently not widely available globally, possibly due to high cost and machine size [28, 37, 48, 61]
	Require specific workforce training and capabilities [27, 45, 56, 58]	Patient's usual care clinicians may not have access to the 3D TBP images, only images via a referral letter. This reduces the benefit of collection of longitudinal images for longer term surveillance [62]
	Manual TBP systems may be more time consuming to perform as it requires imaging of different body regions, especially if the patient has numerous lesions to image [31, 37, 46, 48, 56, 58]	
User perception	There may be a perception among users that photo documentation consumes clinical time, but does not improve early diagnostic [45]	There may be a perception among clinicians that using TBP may lead to more biopsies and greater patient anxiety [50]
	Some perceive the TBP to be costly, especially if used on a regular basis [45]	Some perceive the TBP to be costly, especially if used on a regular basis [45, 50]

Abbreviations: 2D TBP, two‐dimensional total body photography; 3D‐TBP, three‐dimensional total body photography; TBP, total body photography.

^a^ Includes benefits of adopting or implementing 3D TBP, otherwise stated.

^b^ Benefits unique to 3D TBP, compared to 2D TBP.

^c^ Self‐skin examination refers to patients examining their own skin for changes.

^d^ Benefits unique to 2D TBP imaging system, in comparison to 3D TBP.

Four studies collected user feedback and perspectives on the use of TBP and/or AI [36, 48, 50, 51]. Clinicians and dermatologists perceived that augmented intelligence, where AI is used to support clinicians with lesion assessment or diagnostic decision‐making, could improve diagnostic performance (94%) and was more trusted in cases of uncertainty, particularly among less experienced clinicians (76%) [48]. Patients also perceived TBP technologies and their AI capabilities to be trustworthy (88%–93%) and useful (99%) for skin monitoring, and believed that these tools may enhance the performance of dermatologists (88%) [36, 48, 50, 51]. However, Winkler et al. reported that two‐thirds of their patient participants agreed that AI cannot completely replace dermatologist examination [36]. One longitudinal study, conducted over 3 years, reported that 69% of their participants completely trust 3D‐TBP for lesion monitoring and diagnosis at both 18‐ and 36‐month timepoints; however, their level of comfort with the imaging procedure reduced from 71% to 61% by the 36‐month timepoint [50]. Lastly, 93% of participants stated that they were willing to pay a fee to use TBP services, and 71% were accepting of longer examination times if required [36, 50].

Despite these advantages, several challenges were also identified (Table 4). Implementing TBP technologies typically requires substantial resources, including specialized equipment and proprietary software, which can incur significant acquisition and licensing costs [27, 28, 37, 45, 48, 58, 61]. Another consideration is workforce availability and capability, as healthcare professionals may require continuous training to perform TBP and dermoscopic assessments effectively [27, 45, 56, 58]. Limitations related to lesion visibility, due to occlusions (e.g., skin folds, hair) or lack of exposure (e.g., scalp, soles of hands and feet), and patient physical mobility were also noted [28, 29, 36, 38, 42, 45, 47, 49, 50, 54, 61].

## 4 Discussion

This review highlights the evolution and growing role of TBP technologies in skin cancer surveillance. As imaging systems become more advanced and automated, their utility and adoptability can increasingly extend beyond specialized dermatology clinics into broader models of care, including settings where dermatologists are not immediately available. Understanding how these technologies perform in diverse care settings is increasingly important to scale and improve access to skin cancer surveillance and melanoma early detection services.

Australia's clinical practice guidelines recommend periodic full‐body examinations, supported by TBP and dermoscopy, for individuals at high risk of melanoma [65]. These recommendations draw on studies showing that structured surveillance programs can facilitate earlier melanoma detection and may offer favorable cost‐effectiveness [62]. However, availability of TBP technology remains limited. A 2020 survey conducted in Australia found that among 255 general practitioner (GP) and dermatologist respondents, only two generalist GPs (2%), 35 skin cancer GPs (38%), and nine dermatologists (15%), had a TBP technology at their practice. Most of the respondents had to refer their patients for TBP to a private/another dermatologist (23%) or to a private organization (28%) such as MoleMap [66].

Such technologies may be useful for areas where health care access is difficult. For example, Australia's Outer Regional and Remote areas experienced some of the highest age‐standardized melanoma incidence rates and only 6% of the dermatologist workforce were practicing in regional, rural, or remote areas in 2022 [3, 5, 67]. Australians in rural areas may face multiple barriers to melanoma early detection, including constrained health‐seeking capacity and access to specialized skin services, yet very few of the reviewed studies reported on rurality [68, 69].

TBP‐supported skin cancer surveillance services could be integrated into primary care or the community, where it remains the most accessible healthcare services across Australia, as well as into alternative models of care that vary by setting, provider type, or level of clinical support [70, 71]. Telehealth‐enabled TBP models offer particular value by improving access in rural and remote areas, where asynchronous teledermatology can support specialist image review [41, 46, 55, 62]. The increasing use of multidisciplinary teams in primary care expands the potential for TBP‐supported structured skin cancer surveillance, including nurse‐led skin monitoring or standalone imaging services delivered by trained technicians that can be linked to remote dermatology review [72, 73].

Findings from this review indicate that, with appropriate training and service configuration, many TBP‐related tasks, including image acquisition and skin examinations, can be delegated to various healthcare professionals. Image acquisition could be delegated to medical photographers, technicians, or nurses, with training pathways such as MoleMap's programme [35, 39], demonstrating that competency can be built beyond formal nursing or medical training. Where services extend to skin examinations, however, additional training is expected. Australian clinical practice guidelines recommend that clinicians detecting skin cancer be trained in dermoscopy, to improve sensitivity for melanoma detection [74]. Lesion flagging requires understanding of individuals' risks and the ability to recognize morphological structures suggestive of skin cancer [65]. Additional training would improve confidence in conducting full skin examinations and diagnostic accuracy in lesion flagging [71, 74, 75, 76]. Nevertheless, studies have reported high diagnostic accuracy among nurses assessing lesions for referral, supporting the effectiveness of nurse‐led service models [77].

Clinical supervision and decision‐making, in contrast, remain the domain of dermatologists across most studies and care models, reflecting the extensive practical training this role requires. In Australia, dermatologists undergo 4 years of practical training in accredited clinical settings [78]. Skin cancer‐trained GPs typically undergo workshop‐based training ranging from two to more than 7 days, followed by examinations or surgical skills assessment [79]. Dixon et al. found that doctors who had the lowest knowledge scores were those without training or certification in skin cancer management [79]. A lack of comprehensive training may lead to lower diagnostic accuracy, inappropriate and unsafe treatment approaches, or inappropriate specialist referrals [79, 80].

The distinction has practical implications for service design. Nevertheless, Guitera reported that applying a standardized protocol and training from a tertiary service to GP‐led clinic produced comparable clinical outcomes [49]. Several other studies also reported effectiveness of TBP across GP‐ or nurse‐led services, as well as telehealth‐enabled pathways that decouple image acquisition from dermatologist review [30, 39, 41, 46, 49, 55, 62].

User acceptance is also critical, as perceptions of usefulness, effectiveness, trust, and feasibility will shape the long‐term uptake of TBP‐ and/or AI‐supported skin cancer surveillance [81]. Trust, in particular, is central to implementation, as it influences whether healthcare professionals and patients are willing to adopt and continue using new technologies [82, 83]. Factors that contribute to trust in digitalised and AI‐enabled health services include (i) personal elements, such as convenience, usefulness, training, and familiarity; (ii) technological elements, such as accuracy, safety, explainability, privacy, and interoperability; and (iii) institutional elements, including stakeholder engagement, clinician oversight, transparency of use, and impact on communication and workloads [84, 85]. Studies examining user perspectives reported patient trust ratings towards TBP or AI‐enabled TBP ranging from 69% to 93%, and one study reported that 99% of patient participants perceived 3D TBP to be useful [36, 48, 50, 51]. Among dermatologists, Goessinger et al. reported high trust in both 2D and 3D TBP, at 90% and 96% respectively [48]. These findings suggest relatively high levels of patient and dermatologist trust in TBP or AI‐enabled TBP approaches, providing a reasonable foundation for user acceptability to support future implementation efforts. Notably, only two of these studies reported the use of validated questionnaires to measure technology acceptance or trust. Future studies should measure patient and clinician's trust using validated instruments, alongside relevant implementation, service, or patient outcomes such as user perceived usefulness, acceptability, and psychosocial impact [20].

Scaling and implementing TBP technologies in non‐specialized settings as part of a structured skin cancer surveillance program will require careful consideration of workforce capacity, resource availability, workflow integration, and referral pathways. Primary care settings differ greatly from secondary and tertiary settings in terms of staffing, infrastructure, and clinical processes, highlighting the need for tailored implementation strategies that are responsive to resource‐constrained settings and support sustainable adoption. Costs, for example, are a significant barrier to scaling TBP and existing health economic evidence is mixed. Watts et al. found that specialized 2D TBP‐based surveillance can be cost‐saving when melanomas are detected early among high‐risk patients, whereas Lindsay et al. found that 3D TBP‐based surveillance was not cost‐effective compared with usual care, due to device costs [56, 59]. This contrast suggests that the economic case for TBP depends on imaging modality, population, and protocol, and needs further investigation.

### 4.1 Future Directions

As the diagnostic performance of large language models and other AI‐enabled tools in dermatology continues to improve [86, 87], these technologies may help clinicians identify new or changing lesions more consistently and support earlier melanoma detection, while improving workflow efficiency across specialist, primary care, and teledermatology settings [74, 81, 88]. Alongside potential benefits of AI, there are important concerns regarding patient autonomy and informed choice around the use of AI tools, need for formal AI training among clinicians, risk of patient harm in the absence of regulatory standards, ethical risks arising from biased or under‐represented training datasets, or lack of clarity for how legal responsibility should be apportioned if AI contributes to clinical errors [81, 89, 90, 91, 92]. Patient privacy and data security are also of concern, requiring safe and appropriate storage and sharing of images [90, 91]. Overall, standardized governance and ethical framework, or best‐practice guidelines, are needed to ensure AI is implemented responsibly in dermatological care [92, 93].

Reporting and focus on implementation of TBP technologies in real‐world clinical settings remains limited. This gap constrains understanding of how TBP can be scaled and sustained over diverse models of care. Future research would benefit from applying an implementation science lens to investigate the factors and mechanisms that influence adoption, integration, and maintenance of TBP technologies across different healthcare contexts [94, 95]. To support this, future studies can report a minimum set of implementation‐relevant details that operationalize key constructs from established frameworks, such as the Consolidated Framework for Implementation Research (CFIR) [96]. Derived from findings of this review, these could include setting type, staffing model and supervision arrangements, training provided and duration, imaging and process workflow, resources used, and infrastructure or organizational support required. Reporting against these variables would allow for meaningful comparison across settings and provide practical guidance for service planning.

A key limitation of this review was the heterogeneity of study designs and reporting, which limits the comparability of TBP implementation and models of care. Details regarding clinical setting, device types, and workforce roles were at times unclear or inconsistently described, requiring targeted web searches to determine appropriate categorization. It is also likely that TBP is already being used in some primary care settings, particularly skin cancer‐focused or private clinics, which was not fully captured in this review. This may be due to the lack of research conducted in primary care compared with tertiary care or academic affiliated hospitals, where research capacity and resources are typically higher. To a lesser extent, the non‐standardized terminology used for personnel performing TBP may also have contributed to overlap across categories.

## 5 Conclusion

Overall, the included studies illustrate that TBP technologies can be used across different clinical contexts and workflows, and that they are increasingly viewed as feasible and acceptable tools to support structured skin cancer surveillance as these technologies continue to evolve. However, the broader adoption of TBP technologies will depend on how effectively they can be integrated into real‐world clinical settings and workflows, especially at the primary care level where care services are the most accessible to the population. Variability in infrastructure, resources, and workforce capabilities emphasizes the need for context‐specific implementation strategies. Therefore, future research should incorporate an implementation science approach to identify the factors that influence TBP technology adoption, integration, and sustainment across different healthcare settings.

## Funding

NHMRC Centre of Research Excellence (2006551); NHMRC Synergy Grant (2009923). M.J. is supported by NHMRC Fellowship 2034422.

## Conflicts of Interest

H.P.S. is a shareholder of MoleMap NZ Limited and e‐derm consult GmbH and undertakes regular teledermatological reporting for both companies. H.P.S. is a Medical Consultant for Canfield Scientific Inc. and a Medical Advisor for First Derm.

## Supporting information


**Table S1:** Key search terms used across the four databases.
**Figure S1:** Search strategy following Preferred Reporting Items for Systematic Reviews and Meta‐Analyses (PRISMA).
**Figure S2:** Specialist‐led TBP workflow for skin cancer prevention and early detection.
**Figure S3:** Comparative workflow models for TBP delivery—Teledermatology and Nurse‐led pathways.

## Data Availability

The data that support the findings of this study are available from the corresponding author upon reasonable request.
