# Supplementary material for: Implementation of Total Body Photography for Skin Cancer Early Detection: A Systematic Review
Source: Int J Dermatol. 2026 Sep 30;65(11):2266–90. doi: 10.1111/ijd.70679 (PMC13646481; doi:10.1111/ijd.70679)
Supplement: Supplementary file 1 [file IJD-65-2266-s001.docx]

**Supplementary document**

**Table S1.** Key search terms used across the four databases

|  | **PubMed** | **Embase** | **Scopus** | **Web of Science** |
| --- | --- | --- | --- | --- |
| **Total body photography** | ("total body photography"[All Fields] OR "total body imaging"[All Fields] OR "Whole body imaging"[MeSH Terms]) | 'total body photography'/exp OR 'total body photography':ab,ti OR 'whole body imaging'/ exp OR 'whole body imaging' :ab,ti OR tbp:ab,ti OR 'total body imag*':ab,ti OR 'total body photography':ab,ti | ALL(( (tbp OR total AND body AND photography OR total AND body AND imaging OR whole-body AND imaging) | "ALL=(""total body photography"" OR ""whole body imag*"" OR ""total body imag*"")"  "AB=(""total body photography"" OR ""whole body imag*"" OR ""total body imag*"")" |
| AND |  |  |  |  |
| **Skin cancer** | AND ("Skin cancer"[All Fields] OR ("melanoma" [MeSH Terms] OR "melanoma"[All Fields] OR "melanomas"[All Fields] OR "melanoma s"[All Fields]) OR ("naevus"[All Fields] OR "nevus, pigmented"[MeSH Terms] OR ("nevus"[All Fields] AND "pigmented" [All Fields]) OR "pigmented nevus"[All Fields] OR "nevus"[All Fields] OR "nevus" [MeSH Terms]) OR ("naevi"[All Fields] OR "nevus"[MeSH Terms] OR "nevus" [All Fields] OR "nevi"[All Fields]) OR "mole*"[All Fields] OR "lesion*"[All Fields] OR "dermatology"[MeSH Terms] OR "Skin neoplasms"[MeSH Terms]) | 'skin cancer':ab,ti OR melanoma:ab,ti OR naev*:ab,ti OR mole*:ab,ti OR 'skin lesion*':ab,ti OR 'dermatology':ab,ti OR 'skin neoplasm*':ab,ti | (skin AND cancer OR melanoma OR naevus OR naevi OR mole OR lesion OR dermatology OR skin AND neoplasms) )) | "ALL=(""skin cancer"" OR melanoma OR naev* OR nevi OR nevus OR mole OR moles OR ""skin lesion*"" OR dermatology OR ""skin neoplasm*"")"  "AB=(""skin cancer"" OR melanoma OR naev* OR nevi OR nevus OR mole OR moles OR ""skin lesion*"" OR dermatology OR ""skin neoplasm*"")" |
| Filter | English | English | English | English |

**Figure S1.** Search strategy following Preferred Reporting Items for Systematic Reviews and Meta-Analyses (PRISMA)

**Figure S2.** Specialist-led TBP workflow for skin cancer prevention and early detection

**Figure S3.** Comparative workflow models for TBP delivery – Teledermatology and Nurse-led pathways
